# Analysis of Biophysical Variables in an Onion Crop (*Allium cepa* L.) with Nitrogen Fertilization by Sentinel-2 Observations

**DOI:** 10.3390/agronomy12081884

**Published:** 2022-08-11

**Authors:** Alejandra Casella, Luciano Orden, Néstor A. Pezzola, Carolina Bellaccomo, Cristina I. Winschel, Gabriel R. Caballero, Jesús Delegido, Luis Manuel Navas Gracia, Jochem Verrelst

**Affiliations:** 1 Permanent Observatory of Agro-Ecosystems, Climate and Water Institute-National Agricultural Research Centre (ICyA-CNIA), National Institute of Agricultural Technology (INTA), Nicolás Repetto s/n, Hurlingham 1686, Buenos Aires, Argentina; 2 Estación Experimental Agropecuaria INTA Ascasubi (EEA INTA Ascasubi), Ruta 3 Km 794, Hilario Ascasubi 8142, Buenos Aires, Argentina; 3 Centro de Investigación e Innovación Agroalimentaria y Agroambiental (CIAGRO-UMH), GIAAMA Reseach Group, Universidad Miguel Hernández, Carretera de Beniel Km, 03312 Orihuela, Spain; 4 Departamento de Montevideo, Technological University of Uruguay, Av. Italia 6201, Montevideo 11500, Uruguay; 5 Image Processing Laboratory (IPL), Universitat de València, C/Catedrático José Beltrán, 2, 46980 Paterna, Spain; 6 Departamento de Ingeniería Agrícola y Forestal, Escuela Técnica Superior de Ingenierías Agrarias, Universidad de Valladolid, Avenida de Madrid 50, 34004 Palencia, Spain

**Keywords:** vegetation index, LAI, nitrogen, remote sensing, Sentinel-2, precision farming

## Abstract

The production of onions bulbs (*Allium cepa* L.) requires a high amount of nitrogen. According to the demand of sustainable agriculture, the information-development and communication technologies allow for improving the efficiency of nitrogen fertilization. In the south of the province of Buenos Aires, Argentina, between 8000 and 10,000 hectares per year^−1^ are cultivated in the districts of Villarino and Patagones. This work aimed to analyze the relationship of biophysical variables: leaf area index (LAI), canopy chlorophyll content (CCC), and canopy cover factor (fCOVER), with the nitrogen fertilization of an intermediate cycle onion crop and its effects on yield. A field trial study with different doses of granulated urea and granulated urea was carried out, where biophysical characteristics were evaluated in the field and in Sentinel-2 satellite observations. Field data correlated well with satellite data, with an R^2^ of 0.91, 0.96, and 0.85 for LAI, fCOVER, and CCC, respectively. The application of nitrogen in all its doses produced significantly higher yields than the control. The LAI and CCC variables had a positive correlation with yield in the months of November and December. A significant difference was observed between U250 (62 Mg ha^−1^) and the other treatments. The U500 dose led to a yield increase of 27% compared to U250, while the difference between U750 and U500 was 6%.

## 1 Introduction

One of the key challenges in agriculture is to make production systems sustainable. Within this framework, improving the use of fertilizers and decreasing the pollutants in both soil and air involve a long-term reduction in the economic and environmental costs [1]. The proportion of greenhouse gas emissions coming directly from the cultivated areas induced by fertilization is about 23% at a global level [2]. Within the scientific principles of good agricultural practices operates the management of inorganic fertilizers [3]. The basis for good management practices are based on the following principles: suitable source, pertinent dose, specific moment, and precise location [4]. On the other hand, fertilizer-use efficiency reduces production costs. The development of information and communications technology applied to agriculture has allowed increasingly precise study of nitrogen (N) fertilization in farming systems.

The onion crop (*Allium cepa* L.) is an intensive farming system with high fertilizer requirements. This species is the second-most-produced vegetable worldwide, where Argentina stands out among the fresh and dried onion exporters from Latin America [3]. In the valley of the Colorado River, located in the south of Buenos Aires (BVCR), in the Villarino and Patagones districts [5], 8000 ha are sown each year, with an average yield of 45 Mg ha^−1^ [6].

In onion crops, the N uptake is crucial at the beginning of bulb formation, affecting its size and maturity [7]. N must be incorporated into the soil as a fertilizer in order to improve the growth of plant biomass and provide higher chlorophyll content as well [8]. The initial vigor and homogeneity of the emergence are promoted with the N and phosphorus (P) fertilizer combination, and they must be added at seeding. Nevertheless, the N fertilization must be initiated from the third true leaf, with two or three fractional applications. The last one must be implemented when the process of bulbing begins, which is when, along the cropping cycle, the bulb diameter is double the neck diameter [9]. The efficient management of N inputs reduces the negative impact that its leaching in the form of nitrates may have on the environment; it can be a very damaging process for onions grown in sandy soils, which are characterized by low nutrient retention and high permeability [10].

Regarding the monitoring of crop-growth development, the leaf area index (LAI) is defined as the total area of leaves per area of soil [11]. Estimation of LAI provides information about the growth and health of the crops, optimizing the irrigation and plant nutrition processes [12,13]. LAI measures can be accomplished by destructive methods (taking the weight data and the scanned leaves in a known area) or through measurement instruments developed for it such as ceptometers and hemispherical cameras, based on measurement of the radiation intercepted by the vegetation. The accurate management of N fertilizers [14] has boosted the development of diagnostic and estimation systems that consist of using applications (apps) for smartphones such as *PocketLai* with LAI measurement purposes, allowing a simple and economic estimation [15].

The measurement of canopy chlorophyll content (CCC) is an indirect method for evaluating the N content [16,17]. CCC measurement is important because of its function as an indicator of plant-health status, being considered the most relevant vegetal property for estimating productivity [18]. Instruments have been developed for its measurement such as SPAD 502 (*Minolta*), which provides a correlated measure with the amount of chlorophyll per leaf area (Chl).

One of the parameters related to LAI is the calculation of the canopy cover factor (fCOVER). Its monitoring in the agricultural fields gives an indication of the development and vigor of the crop rate [19]. The free-access app *Canopeo* is a fast and accurate tool for calculating this variable. This app quantifies the percentage (%) of the image covered by living (green) vegetation, of any agricultural crop, from photographs and videos obtained with a mobile phone [20].

In recent years, optical remote sensing has been fully used in the estimation of the biophysical parameters, because of its ability to acquire spatial and temporal information on different scales. To monitor croplands with sufficient detail, the spatial resolution must be at least 20 m, and the temporal resolution must be less than one week [21]. This is when the Sentinel-2 (S2) mission of the European Spatial Agency (ESA) becomes important, which represents a spatial resolution up to 10 m and a temporal resolution of five days in Ecuador. Precisely, these satellites have a total of 13 bands, where 4 of them present 10 m of spatial resolution, suitable for the study of intensive agriculture [20], with 6 bands of 20 m and three bands of 60 m for atmospheric correction [22]. The S2 mission is especially important for vegetation studies, thanks to the incorporation of three bands in the red-edge area (705 nm, 740 nm y 783 nm), which allow for establishing an indication series related to N and Chl content. Since, at these lower frequency wavelengths, the energy that can be absorbed by pigments decreases, therefore, a change in reflectance related to the energy absorbed by pigments such as Chl and carotenoids occurs. Within ESA’s free Sentinel Application Platform (SNAP) software, the incorporation of algorithms based on neural-networks (ANNS2) allows the calculation of biophysical variables such as LAI, chlorophyll content at a cover level, and fCOVER in S2 images, among others [23].

Nevertheless, vegetation indices are among the most widely used indicators for the remote sensing of vegetation properties. Probably, the most popular index is the Normalized Difference Vegetation Index (NDVI), originally proposed by Rouse et al. [24], which has been used in several studies about the development of vegetation, Chl, green biomass, N content, and LAI. Apart from NDVI, a wide array of alternative indices was proposed that aimed to optimize sensitivity toward LAI and CCC. Many of these indices are designed with bands in the red-edge area [18,25–27]. The region between 698 nm and 750 nm wavelengths is the spectral zone, where the maximum chlorophyll absorption occurs in red, and the maximum reflexion in NIR is caused by the abundance of the cell structure of the leaf, that is, the LAI and consequently the CCC [28–30]. In this context, several authors used indices that minimize the photosynthetic material effects, such as the MCARI proposed by Daughtry et al. [31], and the TCARI/OSAVI proposed by Haboudane et al. [32], with the aim of minimizing the influence of foliar area and maximizing the sensitivity to Chl.

Recently, several authors analyzed the applicability of S2 data for vegetation properties monitoring studies, especially in the red-edge area. The authors of [33] proposed new indices for these satellites as the Inverted Red-Edge Chlorophyll Index (IRECI), which incorporate the reflectance in four bands of S2 to estimate the CCC parameter, and the S2 red-edge position index (S2REP) was also studied by Clevers and Gitelson [34]. Other studies found good results in agricultural applications by using indices with bands situated in the red-edge area for the estimation of LAI, CCC, and N content [17,27,35].

Given these indices, in order to validate a suitable method capable of systematically providing biophysical variables in any cropland and with the structural characteristics of onion, it is necessary to validate them with the spatial resolution offered by S2 and with measurements throughout the whole growing season coinciding with satellite overpass dates [36].

Field measurements should be performed under different N input conditions, as this will allow us to observe the differences in Chl evolution in different plots and to adjust the indices to obtain Chl maps from satellite images for onion cultivation.

Altogether, the objective of this work is to analyze the application of satellite biophysical indicators to monitor and optimize nitrogen fertilization on the yields of an onion crop. To this end, the products obtained from S2 and vegetation indices will be correlated with the biophysical variables CCC, LAI, and fCOVER obtained with simple-to-use field instruments, and the yield will be compared with different doses of fertilizer.

## 2 Materials and Methods

### 2.1 Study Area and Crop Management

**Figure 1:**
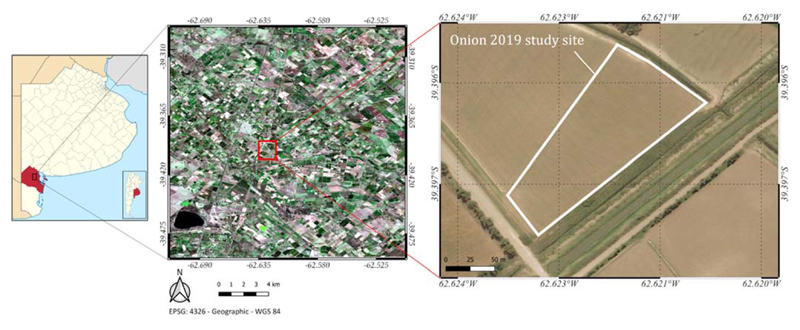
Study site geographic location: Bonaerense Valley of Colorado River in the Villarino district of Buenos Aires province, Argentina. True-color Sentinel-2 image (R = B4, (2 = B3, B = 132) from 13 March 2018, EPSG: 4326—WGS84 (partly adapted from [36]).

The study area is located in Hilario Ascasubi in the Villarino district, Buenos Aires province, in the Bonaerense Valley of Colorado River (BVCR), Argentina (Figure 1). The region is delimited by the 39°10′ and 39° 55′ parallels of south latitude and the 62° 05′ and 63° 55′ meridians of west longitude. It covers a surface of 5304 km^2^, from which 1842 km^2^ are dedicated to crops, most of them under surface irrigation. The main crops are onion (*Allium cepa* L.), maize (*Zea mays* L.), wheat (*Triricum aestivum* L.), sunflower (*Helianthus annuus* L.), alfalfa (*Medicago sativa* L.), wheatgrass (*Thinopyrum* spp.), squash (*Cucurbita* spp.), and potato (*Solanum tuberosum*) [37]. Small-scale establishments predominate. Their size is less than 100 ha, where the onion cultivation is conducted mostly in lots that do not exceed 20 ha.

The BVCR is located in a zone with a temperature steppe semi-arid climate. The average annual temperature is 15 °C, and the average annual precipitation is 483.5 mm [38], with higher precipitations in the spring and summer months. According to soil taxonomy USDA, the type of soils of loam sandy texture from the La Merced series [39], and its physicochemical characteristics has shown initial sampling values of pH 7.8; E.C. 0.70 dS m^−1^; OM 1.35%; Not 0.35; and Pe 13 mg kg^−1^ (determinations were made in Soil and Water Laboratory-EEA INTA Ascasubi). The study area is considered an edaphoclimatic representative of BVCR [40].

For the 2019/2020 season, the experiment was performed in a plot of 2.2 ha, and the type of the studied onion was *Allium cepa* L. cv Torrentina of intermediate day. The preceding summer crop types were sunflower (*Helianthus annuus* L.) (2018/2019) and maize (*Zea mays* L.) (2017/2018).

Mechanical weed control was applied twice before sowing (March and April 2019). Pre-sowing irrigation was accomplished to ensure uniform and rapid germination and seedling emergence. The sowing was conducted mechanically on 3 May 2019, with a seeder for vegetables with mechanized dosage, horizontal axis cylindrical roller, and variable capacity, in 12 lines on 1.2 m wide planks. The seeder was equipped with a fertilizer hopper with dispensers of constant volume and variable speed for the incorporation of granular fertilizer in the sowing line. Moreover, it consists of a hopper for the application of distribution granular insecticides next to the seed. A seed with a germinative power of 86% and 1000-seed weight of 3.7 g were used. The, sowing density was 5 kg ha^−1^. Along with the seed, chlorpyrifos granular insecticide (2.5%) was incorporated at the dose of 7.8 kg ha^−1^, and diammonium phosphate (DAP; 18-46-0) was banded with the seed at planting at 160 kg ha^−1^ laterally to the seeding row.

Five furrow irrigations were performed at different instances of the crop cycle between late August and November. The average quality of irrigation water from the Colorado River in this campaign was pH 8.23; E.C. 1.37 dS m^−1^; and RAS 3.52. In order to control weeds of wide leaves and grasses at two stages of the vegetative development, herbicides were used, and also a manual weeding of the most resistant plant was achieved. Fungicides of systemic and contact action were applied so as to prevent and cure leaf diseases. Concerning fertilization, different doses of urea (46–0–0) were used. The fertilization was performed manually, except after irrigating or prior to rain water on three different occasions (26 August 2019, 24 September 2019, and 25 October 2019). It was applied fractionally from the three true leaves to the bulb formation [41].

### 2.2 Experimental Design

**Figure 2:**
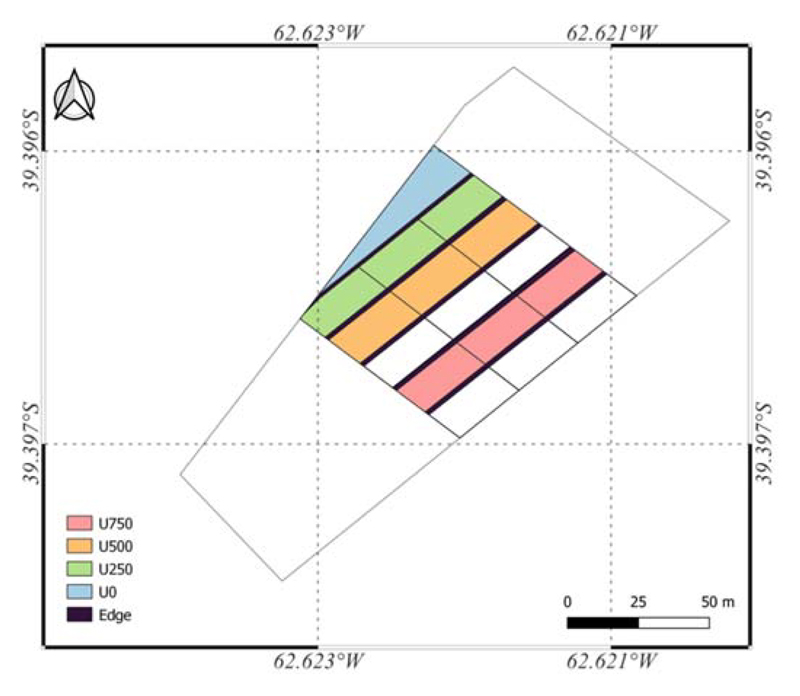
Map of the study area, showingthe location of the experimental site. EPSG: 4326—WGS84.

Three replicates were established of each treatment, composed of three different doses of urea granules (46–0–0), 115 kg IN ha^−1^ (U250), 230 kg N ha^−1^ (U500), and 345 kg ha^−1^ (U750), and a control plot (U0) without N fertilizer (Figure 2). The surface of each treatment was 360 m^2^ (10 planks 1.20 m wide by 30 m long).

The study consisted of measuring biophysical variables LAI, fCOVER, and CCC, utilizing easy-access devices, in an intermediate-cycle onion-crop plot. The field data collection was planned according to the time of fertilization, acquisition dates of the S2 satellite overpass, and crop condition. Five campaigns were made by taking three spatially separated observation points in each sampling unit (Table 1). Finally, the onions were harvested and the output was calculated. Employing the ANNS2 algorithm provided in the SNAP software, the automatic products were calculated and finally, all the results were correlated with the information obtained in the field.

In order to avoid any edge effect [42] we chose a central point at each plot. This was as close as possible to the 10 m by 10 m of the resampling image (Supplementary Materials: Figure S1). The point for the measurements was determined after crop implantation, taking into account the spatial resolution of S2 of 10 m. The most homogeneous pixel of each treatment was then searched for in the S2 image. This pixel was then marked on the ground using GPS, delimiting the study area (Supplementary Materials: Table S1). Field measurements were taken approximately five days before the S2 satellite overpass.

In order to analyze the relation between the obtained biophysical variables and yield, 1 m of the central plank of each plot was harvested (N = 12) on 12 January 2019. The sample bulbs were placed in monofilament plastic net bags, in a place under cover with natural ventilation, to guarantee the drying of the external layers, neck, and root of the bulb (mature). Once the process was completed, a cleaning was performed by extracting dry leaves and roots. The yield obtained was extrapolated for each plot (Mg ha^−1^), counting and weighing each of the harvested bulbs.

#### 2.2.1 In Situ Measurements of Biophysical Variables Data

LAI was measured with the *PocketLai* app [15]. Five observations were taken for each plot, with an average of five interactions (N = 90). In this study, all measurements were made on clear days. Chl values were obtained with the SPAD-502 instrument (*Minolta*), using Equation (1) to perform the calibration [43]. Destructive leaf surface samples were taken from five plants per plot. They were individually identified performing a longitudinal cut of the foliar area of the most-developed sheet in order to obtain the record of six referenced sample points from the base of the apex of each plant (N = 540) [43]. This measurement was not performed in November due to the unavailability of the field instruments at the time of the passing of the satellite. Subsequently, the CCC was calculated with Equation (2).

(1) $$\text{LCC}\left(\mu \text{g}\;{\text{cm}}^{-2}\right)=0.021752\;{\text{SPAD}}^{2.1129}$$

(2) CCC=LCC×LAI

fCOVER was measured with the *Canopeo* app developed for Matlab by Patrignani and Ochsner [20]. The app is based on red-to-green color relations (R/G), blue-to-green colors (B/G), and an excess index of green (2G–R–B). It is used to determine the amount of canopy coverage for the living vegetation of any crop. Measurements were conducted at a height 1.5 m from the ground. In each plot, a photograph was taken, and a sample video of 12 m as a result of the cover percentage shown by the 20 photographs was obtained.

Multitemporal analysis of the relation between the biophysical variables and the cropland yield was estimated, with the objective of determining the moment that has the greatest possibility of predicting performance. Monthly sampled measurements were correlated with the total yield of each parcel per each treatment and per each LAI, CCC, and fCOVER biophysical variable taken, not only in the field but also the ones calculated by the ANNS2 using the S2 images.

#### 2.2.2 Satellite Biophysical Variables Data

Estimation of the biophysical variables and vegetation indices were based on S2 observations [22]. S2 images were acquired on cloud-free dates adjacent to the data collection during the entire phenological cycle (Table 1). The images were downloaded from the ESA server at level 1C. Through the incorporation of Sen2cor complement in the SNAP 7.0 program [44], level 2A images were obtained and corrected atmospherically, which transforms the top of the atmosphere (TOA) reflectance to values of bottom-of-atmosphere (BOA) reflectance. Afterwards, the images were resampled to obtain 10 m pixels in all bands [45]. Finally, the radiometric value was extracted in all bands from the 10 pixels identified in Section 2.2 for field measurements.

Through the Biophysical Operator in SNAP 7.0, LAI (m^2^ m^−2^), fCOVER (%), and CCC (g m^−2^) products for each SU were obtained, selecting only the products in which the quality indicators were optimal. This algorithm uses a neural network for each biophysical variable. Each NN is composed of a layer input of 11 normalized data: B3, B4, B5, B6, B7, B8, B11, B12, cos (zenithal view), cos (solar zenith), and cos (relative azimuth angle), plus a hidden layer with five neurons with tangent sigmoid transfer functions and an output layer with a linear transfer function [23].

#### 2.2.3 Vegetation Indices Measurement

A set of 21 indices, according to the literature the most ordinary ones used for calculating LAI, CCC, and N, was selected (e.g., see reviews in [26,27,29,35]). The indices were calculated using the Band Math tool of SNAP 7.0 software and S2 bands (Table 2).

Biophysical in-field measured variables were correlated with the results generated by the ANNS2 module. Similarly, the field data for each variable were correlated with all vegetation indices. Regression models were adjusted using the September–December data corresponding to the maximum vegetative stage before the bulbification phase. Samples were taken for LAI and fCOVER (N = 40) and for CCC (N = 30).

### 2.3 Statistical Analysis

All analyses were performed using Infostat v.2021 statistical software package linked to R programming [54]. The data-distribution normality of the variables was verified using the Kolmogorov–Smirnov test. The Least Significant Difference (LSD) test was used for multiple comparisons between the means. Analyses of variance at *p* < 0.05 was calculated for all variables over the experiment (ANOVA). Differences between treatments were analyzed using LSD Fisher contrast (*p* < 0.05). The variables were analyzed using a generalized linear mixed model with fertilizer treatment and sampling occasions as fixed factors, while each plot was considered a random factor. Correlations were analyzed by linear regressions of the tracking and crop yield variables.

## 3 Results

### 3.1 Multitemporal Analysis of the Biophysical Variables

**Figure 3:**
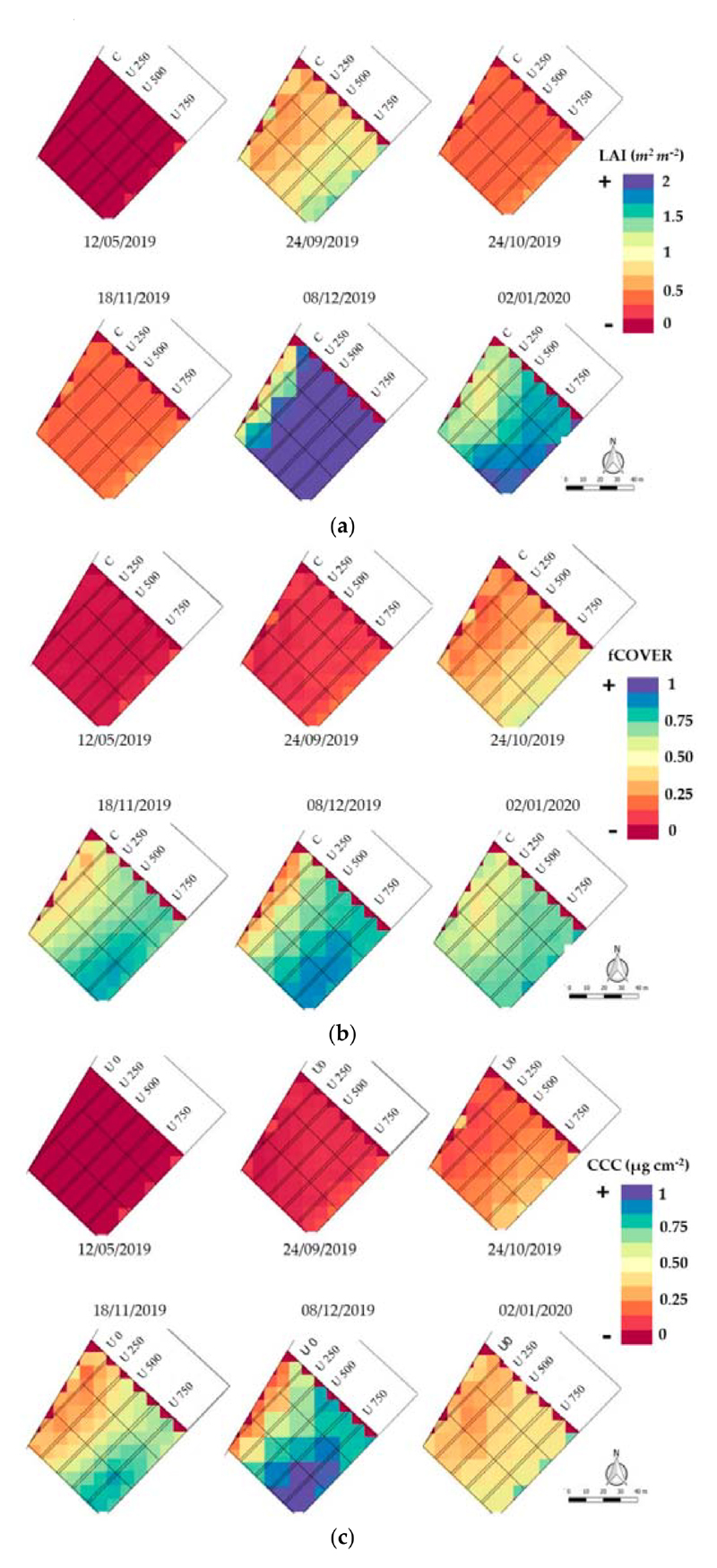
Time series of: (**a**) LAI, (**b**) fCOVER, and (**c**) CCC images during the onion growing cycle. Maps obtained with S2 and SNAP 7.0 software.

With LAI, fCOVER, and CCC products obtained through ANNS2, their multi-temporal evolution during the sampling dates was mapped (Figure 3). Furthermore, the ANNS2 product of each variable was added at the beginning of the growing season (5 December 2019) as a bare-soil reference.

**Figure 4:**
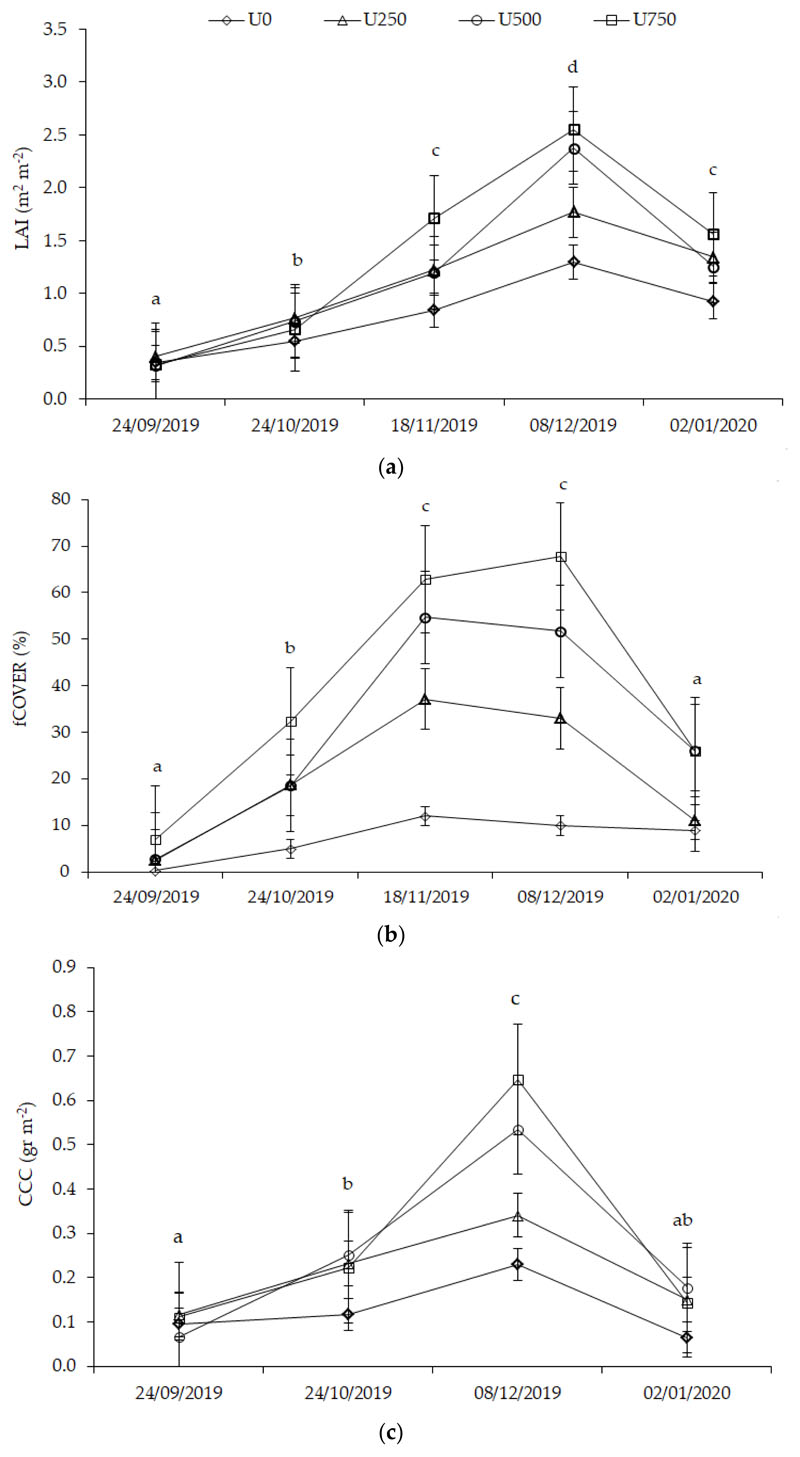
Temporal variation of (**a**) LAI, (**b**) fCOVER, and (**c**) CCC during the onion growing cycle. Lines with different letters represent significant differences between sampling dates at *p* < 0.05. Bars represent the standard error of the mean.

According to the progress of the vegetative development, the parcels with U500 and U750 applications show an index increase from October, with values from 0.4 to 0.8 in November (Figure 4a). From October to November, U750 increase in relation to the other treatments, while U500 grows significantly from November to December, differing from U250 and the control treatment. The maximum value superior to 1.8 is presented in December, approximately to the onset of bulbing, in lots with U750 application. In January, LAI decreased from 0.6 in U0 and U250 to 1.6 in the parcel sectors with U750, close to the harvest.

Highest percentage of the fCOVER value product is found in the parcels with the highest dose of N. Nevertheless, the cover is more homogeneous in treatments with doses of U500 and U750. The values do not exceed 70% in any case and generally do not exceed 64% (Figure 4b). This can be observed in December in a portion of two U750 lots. The fCOVER S2-derived maps (Figure 3b) indicate the differences in coverage between the U0 and the different treatments. The U750 curve grows until December, when it reaches the maximum value, which is when the bulbification begins, and then it declines. It exceeded 60% of coverage from November, reaching a peak of 68% during December. The U500 does also grow until November; it has a slow decrease in coverage at the onset of bulbing and accelerates from December. In all the cases, the fCOVER decreases close to the harvest in January, when the plant loses leaf turgor.

As for CCC, the temporal progress is consistent with the increase in LAI in relation to the N dose applied. It exceeds 0.66 g m^−2^ in lots with U500 and U750 treatments. Values superior to 0.85 g m^−2^ are observed only in a portion of the U750 plots in December (Figure 4c). In January, the variable decreases throughout the field because the photosynthetic process declines toward the end of bulb formation. In the evolution of CCC, a similar behavior to that of LAI is observed. The highest CCC occurs in the month of December in all the treatments. It can be observed that the early stages of the crop lead to similar CCC values, differing from U0. On the other hand, since October, U500 and U750 have both a rapid and sustained increase until December, with a short distance between them. Instead, the distance is greater between U500 and U250, being closer to U0.

### 3.2 Comparison of In Situ Biophysical Variables Measurements and ANNS2 Biophysical Products

**Figure 5:**
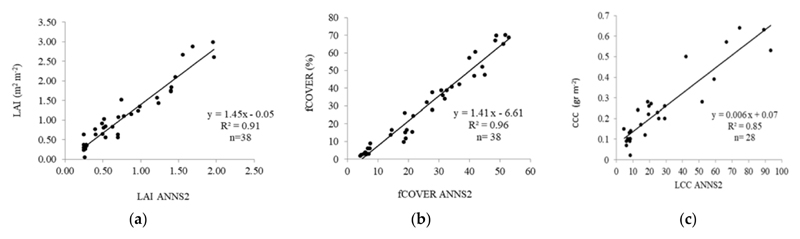
Relationship between (**a**) LAI, (**b**) fCOVER, and (**c**) CCC, based on ANNS2 algorithm for S2 observations and the field experimental data on onion for the same variables, *p*-value < 0.0001.

This section shows the positive linear correlation of the three biophysical variables between the distribution of in situ data points end the data obtained by the ANNS2 algorithm (Figure 5).

For low values, the concentration is higher around the rectangular 1:1 line. fCOVER concentrates a few samples of low values; the others have a more balanced distribution around the 1:1 line that increases the significance of the correlation between both measurements (*p* < 0.05).

### 3.3 Analysis of Vegetation Indices

Linear regresssion models of vegetation indices and biophysical variables were fit (Table 3). It has been observed that the indices that had a better response to estimate LAI were CI_green_ and CI_RedEdget_, with R^2^ = 0.89 and R^2^ = 0.85, respectively. For estimating CCC, IRECI and CI_RedEdge_ responded most sensitively with R^2^ = 0.81. In fact, their sensitivity toward CCC was also demonstrated in maize crops by related studies [55]. Relating to fCOVER retrieval, IRECI and EVI with R^2^ = 0.92 and OSAVI with R^2^ = 0.91 yielded the best results. Table 3 revealed a set of indices with adequate correlation (R^2^ > 0.70) against in situ variables. Lastly, it is notable that ANNS2 yielded superior statistics than the vegetation indices (LAI: R^2^ = 0.91, fCOVER: R^2^ = 0.96, and CCC: R^2^ = 0.85) (Figure 5).

### 3.4 Relationship of the Biophysical Variables and the Yield

**Figure 6:**
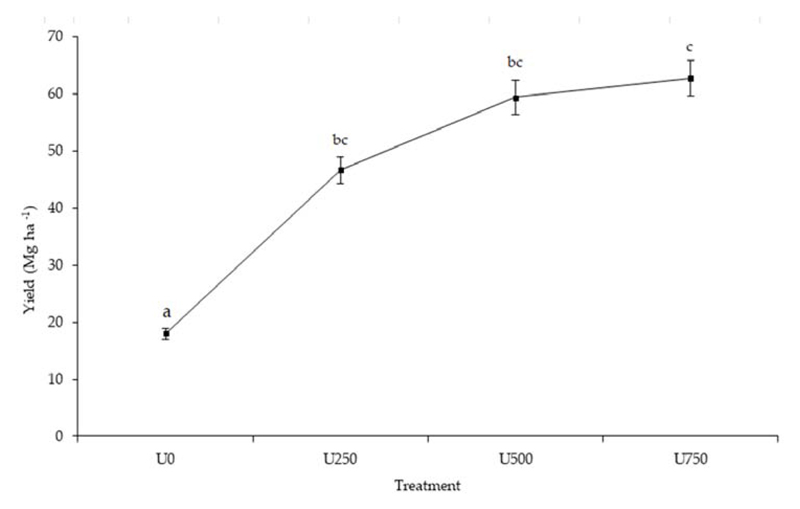
Onion yield (Mg ha^−1^) in different treatments. Lines with different letters represent significant differences at *p* < 0.05. Bars represent the standard error of the mean.

Yield components of the commercial calibers of onion bulbs from each of the plots (N = 12) were analyzed (Mg ha^−1^) (Figure 6), and significant differences were found between the treatments and U0 (p > 0.01). The U500 dose led to a yield increase of 27% compared to U250, while the difference between U750 and U500 was 6%.

Biophysical in-filled collected variables and the S2-derived products were related to parcel yield (Table 4). It was observed that the ANNS2 values improve the information in comparison to the field data. Regarding LAI, the ANNS2 correlation has a slight increase regarding the field data for November and December (R^2^ = 0.53 and R^2^ = 0.70, respectively). Afterwards, LAI decreases in January close to the harvest date (R^2^ = 0.42). The satellite values of CCC show a higher value than the one obtained with the field data in December (R^2^ = 0.69 and R^2^ = 0.23, respectively). In January, they fall, reaching similar values in both measurements (R^2^ = 0.16 and R^2^ = 0.15, respectively). This makes sense from a physiological point of view because, despite having a highly aerial plant mass in the bulb development stage, in the maturation stage the biomass starts to top down. The fCOVER displays the best correlation in November, when the field information improves compared to ANNS2 (R^2^ = 0.84 and R^2^ = 0.66, respectively).

## 4 Discussion

### 4.1 Multitemporal Analysis of the Biophysical Variables

Field-collected data revealed spatial and temporal variability in LAI, fCOVER, and CCC. The decrease in leaf area observed between late November and the beginning of bulbification is due to the fact that the rate of leaf senescence exceeds the rate of leaf-area growth during this period. This agrees with what was reported by Huerres-Perez [56], who indicated that from the beginning of bulb filling, most of the photoassimilates are translocated for bulb development, ceasing the formation of new leaves and causing the physiological senescence of the plant. Thus, at this time, the crop reaches its maximum LAI (Figure 4a). The value of LAI at the beginning of the bulb is one of the variables with the greatest effect on the final crop yield; it is a factor strongly dependent on temperature, water supply, and nutrients at the stage of leaf development and growth; and, basically, it also depends on the duration of this period [41].

In terms of yield, the U0 treatment (24 Mg ha^−1^) is statistically different from the N-fertilized treatments (Figure 6) and is significantly lower than the zonal average yield (45 Mg ha^−1^) [6]. This suggests that N directly influences onion yield, with a direct effect on bulb development and quality [8]. Finally, a significant difference was observed between U250 (62 Mg ha^−1^) and the other treatments, U500 and U750 (79 and 84 Mg ha^−1^, respectively). Onion yield is strictly linked to N fertilization and is directly proportional to the amount of N supplied to the crop [10]. The highest yield was achieved with the U750 treatment, which coincided with the values obtained in onion lots from growers with advanced technology [57]. Only 5 Mg ha^−1^ difference was obtained between the higher dose treatments (U500 and U750), so an increase in N dose affects onion quality, though does not guarantee an increase in yield [58].

High and N deficiency results in yellowing, stunting, twisting, or curling leaves of plants [41]. This coincides with the evolution of the LAI value, which at the beginning of the bulb reaches its maximum (Figure 4a) and then gradually decreases until the bulb matures. Indeed, onion is a crop that shows a quadratic response to increasing fertilizer rates [59], since a first section of fertilizer increase leads to high biomass and bulb yields until a maximum is reached, and then, due to a decrease in fertilizer uptake due to the shallow root depth of the onion, a decrease in biomass occurs [58]. However, according to Tei et al. [60], the relative early growth rate is lower than other crops. This is due to the low canopy-interception capacity of the onion crop, and the low initial-radiation-use efficiency compared to broadleaf crops.

### 4.2 Comparison of In Situ Biophysical Variables Measurements and ANNS2

By using easy-access devices for field measurements on onion croplands, it was possible to validate the automatic products as generated by the ANNS2 algorithms (LAI, CCC, and fCOVER) and correlate these products with the most commonly used vegetation indices. The validation results of the ANNS2 products obtained for LAI (R^2^ = 0.91; *F-anova:* 378.70; *p* < 0.0001), CCC (R^2^ = 0.85; *F-anova:* 146.91; *p* < 0.0001), and fCOVER (R^2^ = 0.96; *F-anova:* 808.70; *p* < 0.0001) outperformed those achieved by the authors of [53], who analyzed ANNS2 on a set of crops in the area. Similar LAI and fCOVER validation results were reported over a variety of croplands in Canada [61] and over wheat fields in northeastern China [62]. Both studies highlighted the qualities of S2 products and, more specifically, of bands in the red-edge domain for the study of the biophysical variables of vegetation.

The spectral resolution of the S2 satellite, integrating the red-edge bands (705 and 740 nm), allowed the evaluation of a diversity of indices potentially sensitive to LAI, CCC, and fCOVER in an erected architecture crop such as onion.

### 4.3 Analysis of Vegetation Indices

Regarding the analyzed indices, LAI had CI_Green_ (R^2^ = 0.89, RSME = 1.22 m^2^ m^−2^) and CI_RedEdge_ (R^2^ = 0.85, RSME = 0.48 m^2^ m^−2^) as the best predictors, showing a better response of the green band of the visible instead of the red edge for this crop, which differs from what was found for wheat [61]. Then, NDRE1 and NDRE2 followed (R^2^ = 0.82, RSME= 1.15 and 1.10 m^2^ m^−2^, respectively), confirming the results obtained by [63] in extensive agriculture, with very similar performance. The LAI indicator, SeLI (R^2^ = 0.82, RSME = 1.09 m^2^ m^−2^), confirms the result obtained by Pasqualotto et al. [35], regarding a set of crop types in orchards in Valencia, Spain, and Foggia, Italy. Moreover, the correlations between RVI (R^2^ = 0.81, RSME = 2.31 m^2^ m^−2^) and NDVI (R^2^ = 0.81, RSME = 0.93 m^2^ m^−2^) were satisfactory.

The vegetation indices that predicted CCC best were IRECI (R^2^ = 0.81, RSME = 0.11 g m^−2^) and CI_RedEdge_ (R^2^ = 0.81, RSME = 0.63 g m^−2^). This is in agreement with the results obtained by the authors of [33], who determined that IRECI was an almost direct estimate of canopy chlorophyll content, measured in the field through the use of S2 observations, while other authors [62] obtained good correlations with CI_RedEdge_ in a diverse group of crops: in potato [28] and for maize and soybean [33]. Regarding the correlation between NDRE1 (R^2^ = 0.80, RSME = 0.17 g m^−2^) and OSAVI (R^2^ = 0.80, RSME = 0.12 g m^−2^), the correlation was improved with field data obtained in the conifer canopy [64].

Regarding LAI and CCC, superior correlations were achieved with indices equipped with bands in the red-edge region (600–750 nm). This region is strongly influenced by the amount of chlorophyll and, consequently, by green LAI, which was confirmed by Delegido et al. [27]. For chlorophyll analysis in the canopy, vegetation indices modified with the incorporation of the 705 and 740 nm S2 bands achieved the best results. For LAI, these red-edge indices outperform NDVI and the vegetation index with a visible domain component, as previously reported by [62].

Concerning fCOVER response, OSAVI, IRECI, and EVI (R^2^ > 0.90) produced similar results, with IRECI showing the lowest RMSE (0.10). Moreover, NDRE 1, NDRE 2, CI_RedEdge_, TRBI, and NAOC led to similar performance, with R^2^ > 0.85 and RMSE < 0.62%, except for NAOC (RSME = 1.12%). Therefore, for this biophysical variable, the results obtained with the vegetation indices with bands in the red-edge domain showed no difference compared to the vegetation index with bands in the visible spectrum.

An interesting result was observed in the response to MCARI and TCARI, which showed a strong linear correlation with LAI (R^2^ = 0.63 and R^2^ = 0.65, respectively, *p* < 0.0001) and CCC (R^2^ = 0.70 and R^2^ = 0.64, respectively, *p* < 0.0001), but when combined with OSAVI, the R^2^ value decreased for LAI and for CCC. The results do not coincide with studies carried out in soybean, maize, and potato [28,34] but are in agreement with those obtained by Zarco-Tejada et al. [64] in pine forests in the region of Extremadura, Spain. The TCARI–OSAVI index responded negatively for CCC concentrations below 0.15 g m^−2^, which could be due to the background effect rather than differences in CCC [63]. This may be due to the fact that the leaf structure of the onion plant does not completely cover the soil. On the other hand, given that the vegetation indices respond to different lighting conditions, soil type, management differences, and environmental variability, it will be necessary to adjust the new models for their generalized application to onion crops.

## 5 Conclusions

This study demonstrated the potential of the simple and cost-effective developed methodology for the timing and rate of N-fertilization anticipation over the onion crop in an irrigated extensively cultivated area in the south of Buenos Aires province, Argentina. The availability of easily accessible tools for farmers will allow better crop monitoring and, thus, optimization of yields. The SNAP’s LAI, CCC, and fCOVER products, based on S2 spectral data and neural networks, proved to outperform the vegetation indices at the retrieval stage of these biophysical variables and to be suitable for monitoring the intensive production of a leaf-structured crop such as *Allium cepa* L.

In situ measured biophysical variables showed a strong correlation with the estimates in the study site. The analyzed vegetation indices can provide useful information to predict onion biophysical variables such as LAI, CCC, and fCOVER (R^2^ > 0.7 in general terms) and be a proxy for the plant’s N-content determination.

In terms of yield, it was confirmed that applying N in the form of urea granulates to intermediate-day onion crops boosts crop yield. As a non-significant difference in yield between the doses, U500 and U750, was found, this research presents an opportunity to address fertilizer-dose optimization, which will help to reduce economic and environmental costs.

We firmly suggest complementing this study with the analysis of N and Chl in plant tissue samples, to obtain more accurately adjusted models based on the S2 spectral information.

## Supplementary Material

The following supporting information can be downloaded at: https://www.mdpi.com/article/10.3390/agronomy12081884/s1. Figure S1: Map of the study area, showing the location of the experimental site (Hilario Ascasubi, Buenos Aires province, Argentina) with the sample points. Table S1: Geopositioning of sampling points in the field experimental design.

Supplementary Material

## Figures and Tables

**Table 1:** Sampling of biophysical variables in onions and ephemerides from S2 images (Hilario Ascasubi, Buenos Aires, Argentina 2019).

Sampling	Date Field	Satellite	Phenological Stage	
1	25/09/2019	24/09/2019		4–6 leaves
2	24/10/2019	24/10/2019	Vegetative	6–8 leaves
3	15/11/2019	18/11/2019		8–10 leaves
4	06/12/2019	08/12/2019	Bulbification	
5	06/01/2020	02/01/2020	Pre-harvest	

**Table 2:** Calculated vegetation indices. R represents the reflectance in the wavelength λ. It shows the formula with the bands corresponding to the S2 satellite.

Index	Formula	S2 Formula	Reference
CI_Green_	$\frac{{\text{R}}_{783}}{{\text{R}}_{560}}-1$	$\frac{\text{B7}}{\text{B3}}-1$	[46]
CI_RedEdge_	$\frac{{\text{R}}_{783}}{{\text{R}}_{705}}-1$	$\frac{{\text{B}}_{7}}{{\text{B}}_{5}}-1$	[46]
EVI	2.5 (R_842_ – R_665_)/(R_842_ + 6 R_665_–7.5 R_490_ + 1)	2.5 (B8 – B4)/(B8 + 6 B4 – 7.5 B2 + 1)	[46]
EVI	2.^5^ (R_842_ – R_665_)′ (R_842_ + 6 R_665_ – 7.5 R_490_ + 1)	2.5 (B8 – B4)/(B8 + 6 B4 – 7.5 B2 + 1)	[47]
GNDVI	$\frac{{\text{R}}_{842}-{\text{R}}_{560}}{{\text{R}}_{842}+{\text{R}}_{560}}$	$\frac{\text{B}8-\text{B3}}{\text{B}8+\text{B3}}$	[25]
IRECI	$\left({\text{R}}_{783}-{\text{R}}_{665}\right)/\frac{{\text{R}}_{705}}{{\text{R}}_{740}}$	$\left(\text{B7–B4}\right)/\frac{\text{B5}}{\text{B6}}$	[33]
MCARI	[(R_705_ – R_665_) – 0.2 (R_705_ – R_560_)](R_705_/R_665_)	[(B5 – B4) – 0.2(B5 – B3)](B5/B4)	[31]
MCARI-OSAVI	[(R_705_ – R_665_) – 0.2 (R_705_ – R_560_)](R_705_/R_665_)/[(1 + 0.16)(R_740_ – R_705_)/(R_740_ + R_705_ + 0.16)]	[(B5 – B4) – 0.2 (B5 – B3)](B5/B4)/[(1 + 0.16) (B6 – B5)/(B6 + B5 + 0.16)]	[26]
MTCI	$\frac{{\text{R}}_{740}-{\text{R}}_{705}}{{\text{R}}_{740}+{\text{R}}_{665}}$	$\frac{{\text{B}}_{6}-{\text{B}}_{5}}{{\text{B}}_{5}-{\text{B}}_{4}}$	[30]
NAOC	$1-\frac{\int_{665}^{783}\cdot \text{Rd}\lambda }{\left({\text{R}}_{783}-{\text{R}}_{665}\right)}$	$1-\frac{\int_{\text{B4}}^{\text{B7}}\cdot \text{Rd}\lambda }{\left(\text{B7}-\text{B4}\right)}$	[43]
NDI45	$\frac{{\text{R}}_{705}-{\text{R}}_{665}}{{\text{R}}_{705}+{\text{R}}_{665}}$	$\frac{\text{B5}-\text{B4}}{\text{B5}+\text{B4}}$	[29]
NDRE1	$\frac{{\text{R}}_{740}-{\text{R}}_{795}}{{\text{R}}_{740}+{\text{R}}_{705}}$	$\frac{\text{B6}-\text{B5}}{\text{B6}+\text{B5}}$	[25]
NDRE2	$\frac{{\text{R}}_{783}-{\text{R}}_{705}}{{\text{R}}_{783}+{\text{R}}_{705}}$	$\frac{\text{B7}-\text{B5}}{\text{B7}+\text{B5}}$	[48]
NDVI	$\frac{{\text{R}}_{842}-{\text{R}}_{665}}{{\text{R}}_{842}+{\text{R}}_{665}}$	$\frac{\text{B8}-\text{B4}}{\text{B8}+\text{B4}}$	[24]
OSAVI	(1 + 0.16)(R_740_ – R_705_) / (R_740_ + R_705_ + 0.16)	(1 + 0.16) (B6 – B5)/(B6 + B5 + 0.16)	[49]
RENDVI	$\frac{{\text{R}}_{842}-{\text{R}}_{740}}{{\text{R}}_{842}+{\text{R}}_{740}}$	$\frac{\text{B8}-\text{B6}}{\text{B8}+\text{B6}}$	[50]
RVI	$\frac{{\text{R}}_{842}}{{\text{R}}_{665}}$	$\frac{{\text{B}}_{8}}{{\text{B}}_{4}}$	[51]
S2-REP	$705+35\left[\frac{\frac{\text{R783-R665}}{2}-705}{740-705}\right]$	$705+35\left[\frac{\frac{\text{B7–B4}}{2}-705}{740-705}\right]$	[33]
SeLI	$\frac{{\text{R}}_{865}-{\text{R}}_{705}}{{\text{R}}_{865}+{\text{R}}_{705}}$	$\frac{\text{B8a}-\text{B5}}{\text{B8a}+\text{B5}}$	[52]
TCARI	3[(R_740_ – R_705_) – 0.2 (R_740_ – R_560_)(R_740_/R_705_)]	3 [(B6 – B5) – 0.2 (B6 – B3) (B6/B5)]	[32]
TCARI-OSAVI	3[(R_740_ – R_705_) – 0.2(R_740_ – R_560_)(R_740_/R_705_)]/(1 + °.1.6)(R_740_ – R_705_)/(R_740_+R_705_ + 0.16)	3 [(B6 – B5) – 0.2 (B6 – B3) (B6/B5)]/(1 + 0.16) (B6 – B5)/(B6 + B5 + 0.16)	[32]
TRBI	$\frac{{\text{R}}_{560}+{\text{R}}_{665}}{{\text{R}}_{842}}$	$\frac{\text{B3}+\text{B4}}{\text{B8}}$	[53]

**Table 3:** Linear regression statistics between the vegetation indices and biophysical variables.

Index	*R* ^2^	LAI (m^2^ m^−2^) *F-anova*	*p*-Value	*R* ^2^	CCC (g m^−2^) *F-anova*	*p*-Value	*R* ^2^	fCOVER (%) *F-anova*	*p*-Value
CI_green_	0.89	293.48	***	0.78	98.62	***	0.77	125.32	***
CI_RedEdge_	0.85	215.54	***	0.81	117.83	***	0.85	216.91	***
EVI	0.59	55.32	***	0.78	101.24	***	0.92	420.06	***
GNDVI	0.81	158.63	***	0.56	33.91	***	0.80	151.61	***
IRECI	0.71	94.20	***	0.80	113.73	***	0.92	415.27	***
MCARI	0.64	66.16	***	0.70	66.62	***	0.80	151.46	***
MOSAVI	0.00	0.0005	ns	0.00	0.0003	ns	0.04	1.54	ns
MTCI	0.25	12.58	**	0.53	30.70	***	0.29	15.85	***
NAOC	0.79	145.35	***	0.76	88.51	***	0.86	238.77	***
NDI45	0.79	142.36	***	0.58	38.02	***	0.76	121.04	***
NDRE1	0.82	171.33	***	0.79	108.38	***	0.88	281.44	***
NDRE2	0.82	169.11	***	0.79	104.77	***	0.87	257.74	***
NDVI	0.80	156.82	***	0.78	102.14	***	0.89	305.70	***
OSAVI	0.77	1.20	***	0.79	108.19	***	0.91	393.07	***
RENDVI	0.41	28.68	***	0.50	28.52	***	0.69	85.65	***
RVI	0.81	157.67	***	0.77	94.68	***	0.77	116.82	***
S2-REP	0.21	10.13	**	0.22	7.84	**	0.27	13.90	***
SeLI	0.82	178.61	***	0.79	183.02	***	0.86	236.48	***
TCARI	0.45	31.13	***	0.64	49.43	***	0.708	135.84	***
TOSAVI	0.07	3.03	ns	0.09	2.72	ns	0.20	9.56	**
TRBI	0.69	84.45	***	0.74	80.78	***	0.89	301.15	***

** and *** significant at *p*-value < 0.001, and < 0.0001, respectively. ns = not significant at *p* < 0.05.

**Table 4:** Relationship of the LAI, fCOVER, and CCC variables of the in situ obtained data and the ANNS2-obtained vegetation products, for each date, with onion yields (Mg ha^−1^).

Date		Field			Satellite		
		LAI (m^2^ m^−2^)	fCOVER (%)	CCC (g m^−2^)	LAI (m^2^ m^−2^)	fCOVER %	CCC (g m^−2^)
	R^2^	0.06	0.22	0.00	0.00	0.01	0.00
24/09/2019	*p-value*	ns	ns	ns	ns	ns	ns
	*F-anova*	0.52	2.22	0.004	0.0003	0.08	0.004
	R^2^	0.19	0.26	0.15	0.10	0.23	0.24
24/10/2019	*p-value*	ns	ns	ns	ns	ns	ns
	*F-anova*	1.97	2.77	1.45	0.93	2.36	2.55
	R^2^	0.52	0.84	-	0.53	0.66	-
18/11/2019	*p-value*	*	***	-	*	**	-
	*F-anova*	8.54	41.25	-	9.20	15.50	-
	R^2^	0.61	0.72	0.23	0.70	0.66	0.69
08/12/2019	*p-value*	**	**	ns	**	**	**
	*F-anova*	12.36	18.08	2.34	18.72	15.21	18.10
	R^2^	0.45	0.18	0.04	0.35	0.13	0.05
02/01/2020	*p-value*	*	3.09	1.44	*	2.43	1.55
	*F-anova*	8.31	ns	ns	5.89	ns	ns

*, **, and *** significant difference among treatments at *p*-value < 0.01, < 0.001, and < 0.0001, respectively. ns: no significant difference.

## Data Availability

Not applicable.
