# Supplementary material for: Analysis of Biophysical Variables in an Onion Crop (Allium cepa L.) with Nitrogen Fertilization by Sentinel-2 Observations
Source: Agronomy (Basel). Author manuscript; Available in PMC 2022 Sep 7. (PMC7613392; doi:10.3390/agronomy12081884)
Supplement: Supplementary Material [file EMS152691-supplement-Supplementary_Material.pdf]

Supplementary Materials

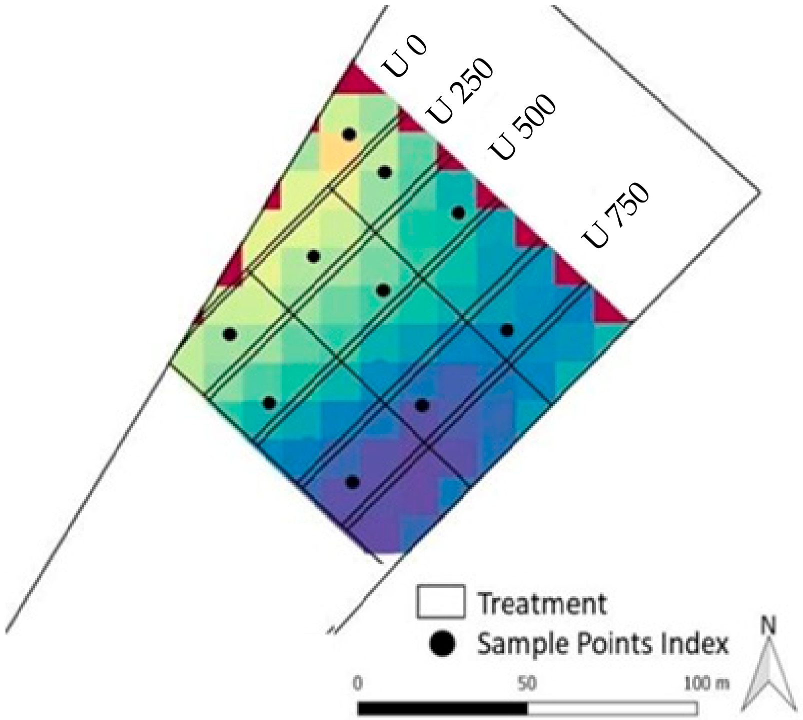

Figure S1. Map of the study area, showing the location of the experimental site (Hilario Ascasubi, Buenos Aires province, Argentina) with the sample points.

Table S1. Geopositioning of sampling points in the field experimental design.

Treatment Sample Point	Pixel-X	Pixel-Y	Longitude	Latitude
U 0	35.5	25.5	-62,62,215,748,609,030	-39,39,585,841,554,580
U 250	36.5	26.5	-62,622,040,868,071,300	-39,39,594,814,381,910
U 250	34.5	28.5	-62,622,272,160,128,200	-39,39,612,910,902,470
U 250	32.5	30.5	-62,62,250,345,338,010	-39,39,631,007,377,070
U 500	38.5	27.5	-62,62,180,811,765,850	-39,39,603,749,454,560
U 500	36.5	29.5	-62,62,203,940,940,340	-39,39,621,846,020,960
U 500	33.5	32.5	-62,622,386,349,261,700	-39,39,648,990,784,380
U 750	39.5	30.5	-62,621,690,525,546,600	-39,39,630,743,337,790
U 750	37.5	32.5	-62,62,192,181,787,160	-39,39,648,839,927,020
U 750	35.5	34.5	-62,622,153,111,391,800	-39,39,666,936,470,270
